# Supplementary material for: A Genome-Wide Association Study of Protein, Oil, and Amino Acid Content in Wild Soybean (Glycine soja)
Source: Plants (Basel). 2023 Apr 16;12(8):1665. doi: 10.3390/plants12081665 (PMC10143452; doi:10.3390/plants12081665)
Supplement: Supplementary file 1 [file plants-12-01665-s001.zip › Figure S1.pdf]

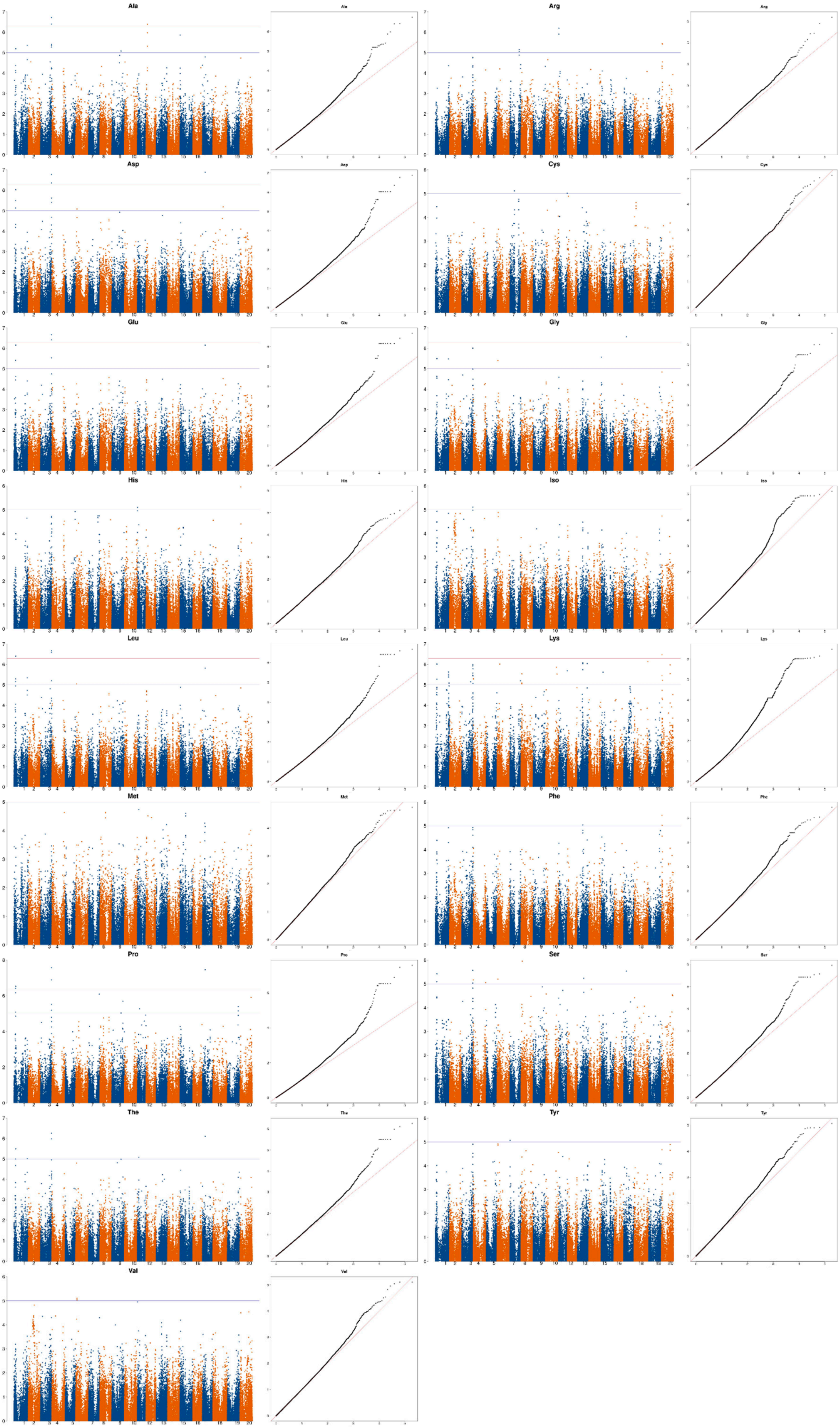
Ala
Arg
Asp
Cys
Glu
Gly
His
Iso
Leu
Lys
Met
Phe
Pro
Ser
The
Tyr
Val

Figure S1. Manhattan plots and quantile–quantile (QQ) plots for 17 amino acid content in 203 wild soybean accessions. In the Manhattan plots, the blue line indicates the genome-wide threshold -$\log_{10}(P)$ = 4.98, and the red line represents -$\log_{10}(P)$ = 6.29, calculated using the Bonferroni method.
